# Lightweight Fine-Grained Access Control for Wireless Body Area Networks

**DOI:** 10.3390/s20041088

**Published:** 2020-02-17

**Authors:** Mohammad Ali, Mohammad-Reza Sadeghi, Ximeng Liu

**Affiliations:** 1 Department of Mathematics and Computer Science, Amirkabir University of Technology, Tehran 159163-4311, Iran; mali71@aut.ac.ir (M.A.); msadeghi@aut.ac.ir (M.-R.S.); 2 College of Mathematics and Computer Science, Fuzhou University, Fuzhou 350108, China; 3 Guangdong Provincial Key Laboratory of Data Security and Privacy Protection, Guangzhou 510632, China; 4 Shaanxi Key Laboratory of Network and System Security, Xidian University, Xi’an 710071, China

**Keywords:** fine-grained access control, wireless body area networks, lightweight computation, attribute-based encryption, cloud computing

## Abstract

Wireless Body Area Network (WBAN) is a highly promising technology enabling health providers to remotely monitor vital parameters of patients via tiny wearable and implantable sensors. In a WBAN, medical data is collected by several tiny sensors and usually transmitted to a server-side (e.g., a cloud service provider) for long-term storage and online/offline processing. However, as the health data includes several sensitive information, providing confidentiality and fine-grained access control is necessary to preserve the privacy of patients. In this paper, we design an attribute-based encryption (ABE) scheme with lightweight encryption and decryption mechanisms. Our scheme enables tiny sensors to encrypt the collected data under an access control policy by performing very few computational operations. Also, the computational overhead on the users in the decryption phase is lightweight, and most of the operations are performed by the cloud server. In comparison with some excellent ABE schemes, our encryption mechanism is more than 100 times faster, and the communication overhead in our scheme decreases significantly. We provide the security definition for the new primitive and prove its security in the standard model and under the hardness assumption of the decisional bilinear Diffie-Hellman (DBDH) problem.

## 1. Introduction

Nowadays, because of several improvements in public health, nourishment, and medicine, the aging population around the world has been quickly increasing. For instance, in the United States, the population of people over the age of 65 is predicted to double by 2040 [1]. Also, in the People’s Republic of China, it is predicted that the number of people aged over 60 will be doubled by 2040 [2]. These estimates show that increasing the number of elderly people with various health problems may significantly increase healthcare costs in the near future [3,4,5]. Therefore, the current healthcare system may not be able to respond to the patients’ requests in the coming years [4,6].

**Figure 1:**
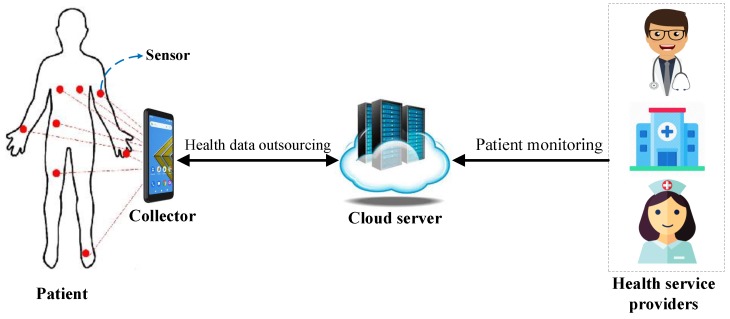
A typical WBAN.

With the rapid development of medical sensors and wireless communications [7], wireless body area networks (WBANs) are under rapid development. WBANs have significant potential for improving the current health system. As we have shown in Figure 1, a WBAN consists of several implantable or wearable sensors and a controller. The responsibility of the sensors is to monitor the vital parameters of a patient (e.g., breathing rate, blood pressure, diabetes, and asthma) as well as measuring the environmental parameters such as humidity and temperature. The sensors collect health data files and encrypt them. Then, they transfer the generated ciphertext to the collector. The controller working as a gateway transfers the gathered health data to a cloud service provider. WBANs can significantly raise the efficiency of healthcare services as individuals do not need to visit the hospital anymore. Thus, WBANs play an important role in affording highly reliable ubiquitous healthcare services. However, as in the cloud-based WBANs the health data are outsourced to a third-party cloud server, some security concerns over fine-grained access control and data confidentiality are raised. Moreover, as tiny sensors in WBANs usually have limited computational and power resources, providing a secure lightweight encryption mechanism is another challenge in this scenario.

Attribute-based encryption (ABE) [8,9] is a promising tool to afford confidentiality and fine-grained access control simultaneously. Generally, ABE schemes can be divided into three categories key-policy ABE (KP-ABE) [10], ciphertext-policy ABE (CP-ABE) [11], and dual-policy ABE (DP-ABE) [12]. In a KP-ABE, a data user’s secret-key is associated with an access control policy which is defined by a central authority, and each ciphertext is labeled by a set of attributes. A data user can decrypt a ciphertext if the access policy associated with its secret-key is satisfied by the attribute set associated with the ciphertext. Also, in a CP-ABE, a data user’s secret-key is associated with the data user’s attributes, and ciphertexts are associated with an access control policy. The secret-key of a data user can decrypt a ciphertext only if the attribute set of the data user satisfies the access policy associated with the ciphertext. In a DP-ABE scheme, secret-key of a data user corresponds to both an access control policy defined by the central authority and the data user’s attributes. Each ciphertext also is associated with both an access control policy defined by a data owner and a set of attributes. A data user can decrypt a ciphertext if and only if the access control policy embedded in the ciphertext is satisfied by attributes of the data user, and attributes of the ciphertext satisfy the data user’s access policy. It seems that CP-ABE is more comfortable for both data owners and data users.

However, to the best of the authors’ knowledge, current ABE schemes suffer from expensive computational operations in the encryption phase. Therefore, since the sensors have limited computational and power resources, existing ABE schemes are not appropriate for providing fine-grained access control in WBANs. To address this problem, in this paper, we design a lightweight fine-grained access control scheme called LW-FGAC which is able to offer lightweight encryption and decryption mechanisms. Our main contributions are given below:

- Lightweight encryption mechanism: Our proposed encryption mechanism is very efficient. In fact, in contrast with existing schemes, in our encryption scheme, the number of expensive operations performed by data owners (smart devices in the WBAN) does not depend on the number of attributes in the access control policy, and almost all the computational operations are offloaded onto the cloud service provider. As we will see, our encryption approach is more than 100 times faster than some excellent schemes in the literature.
- Lightweight communication overhead: In LW-FGAC, in comparison with the existing work, the communication overhead from a data owner to the cloud server is very few. Indeed, in LW-FGAC, lightweight partial ciphertexts are uploaded to the cloud server instead of ciphertexts with huge size.
- Lightweight decryption mechanism: Similar to the encryption phase, in the decryption phase, heavy computational operations can be outsourced to the CSP such that the CSP learns no partial information about data users’ secret-keys and also the underlying data files.
- Security definition and security proof: We formalize the system model and the security definition for the new primitive. Also, we prove the security of the scheme under the hardness assumption of the DBDH problem in the standard model.

## 2. Related Work

Cao et al. presented a thorough survey on WBANs [13]. Their work surveyed several basic WBAN research projects and enabling technologies. It also explored application scenarios, radio systems, smart devices, and the interconnection of WBANs to afford perspective on the trade-offs between data rate, power consumption, and network coverage. Li et al. [14] introduced an anonymous key agreement and mutual authentication scheme for WBANs. Their work enables the sensor nodes attached to patients’ bodies to authenticate with the local server and establish a session key in an unlinkable and anonymous way. Chen et al. presented a detailed review of body area networks and their related issues [15]. They provided a comprehensive investigation of sensor devices, data link layer, physical layer, and radio technology aspects of WBANs. They also introduced some of the design challenges and open problems in this area. Zhang et al. [16] designed an efficient key agreement mechanism for WBANs. Their scheme enables neighboring nodes in WBANs to share a common key established by electrocardiogram (ECG) signals. Their proposed key agreement scheme can secure data communications over WBANs in a plug-n-play manner with no key distribution overhead. He et al. [17] introduced the security and performance challenges related to sensor networks for wireless medical monitoring. They also proposed an attack-resistant and lightweight trust management scheme. Zhou et al. [18] presented several fundamental and sophisticated cyberattacks to wireless sensors networks and introduced some substantial and promising solutions to satisfy the requirements. Ghamari et al. [19] presented a survey on WBANs for health care systems. They compared some current low-power communication technologies supporting the quick advancement and deployment of WBANs. Zhou et al. [20] proposed a privacy-preserving key management system for cloud-based WBANs in m-healthcare social networks. Their proposed scheme protects the patient’s identity privacy, location privacy, and sensor deployment privacy by employing a blinding technique and embedding the human body’s symmetric structure into the Blom’s symmetric-key mechanism with a modified secret sharing technique. Liu et al. [21] designed a medium access control for WBANs. In their work, by employing the Nash Bargaining Solution (NBS), they proposed a cooperative game-theoretic method providing priority-based tuning and maintaining the fairness axioms of game theory. Shen et al. [22] proposed a lightweight multi-layer authentication protocol for WBANs. In their work, using the ECC algorithm, they designed a one-to-many group authentication mechanism and a group key establishment algorithm between personal digital assistants and the other sensor nodes. They also designed a certificateless authentication mechanism without pairing. Whereas, it is known that access control is a major problem in WBANs [23], the mentioned schemes did not consider this problem.

ABE is a promising solution to the access control problem. The notion of ABE was first proposed by Sahai and Waters [8]. In their proposed scheme, a data owner can determine the authorized user to access its data by specifying an attribute set and a threshold value *d*. Each data user that has at least *d* common attributes with the specified set can access the outsourced data. After proposing ABE schemes, three schemes [12,24,25] divided ABE schemes into three categories key-policy ABE (KP-ABE), ciphertext-policy ABE (CP-ABE), and dual-policy ABE (DP-ABE), respectively. Zhou et al. [26] designed a constant size CP-ABE. In their work, the size of ciphertexts is not sensitive to the number of attributes in access control policies. This feature significantly reduces the storage and communication overhead of the system. Guo et al. [27] designed a lightweight CP-ABE scheme with a constant secret-key size [28]. In their scheme, the length of a user’s secret-key does not depend on the number of the user’s attributes. Chen et al. [29] proposed an attribute-based scheme with short ciphertexts and signatures. Their proposed scheme has adaptive security in the standard model. However, none of the schemes presented in [26,28,29] provide a flexible access structure. Indeed, the schemes presented in [26,28] only supports the And-gates access control policy, and [29] only provides the threshold access control policy. Yao et al. [30], designed a KP-ABE scheme for IoT applications. Their work supports access trees as access control policies. Also, in their work, by using the ECC algorithm, the communication and storage overhead is reduced significantly. He et al. [31] proposed an ABE scheme for mobile cloud-assisted cyber-physical systems. In their work, by eliminating pairing operations, they tried to lighten the encryption and decryption overhead. However, several expensive operations still remain. So, it seems that their scheme is not suitable for WBANs. Moreover, none of the mentioned ABE schemes provide lightweight encryption and decryption mechanisms which is not desirable for WBANs. To address this issue, several lightweight ABE schemes have been put forward. Yang et al. [32,33] designed lightweight access control systems for healthcare IoT networks. Their scheme provides a lightweight decryption mechanism and supports access trees as access control policies. Also, their schemes have adaptive security in the standard model. Xu et al. [34] proposed a lightweight DP-ABE for healthcare IoT systems. Their work offers a lightweight decryption system, and it is provably secure in the selective model. Lin et al. [35] proposed CP-ABE with a lightweight decryption mechanism by using an outsourcing technique. Lai et al. [36] put forward a CP-ABE scheme with verifiable outsourced decryption. Their work also provides a lightweight decryption approach and is provable in the adaptive model. However, none of the mentioned ABE schemes provide a lightweight encryption mechanism. Indeed, in these schemes, the computational operations on the user’s side in the encryption phase is very expensive. This feature definitely makes such schemes inappropriate for WBANs. Table 1 compares the features of the mentioned ABE schemes with our proposed LW-FGAC. As we see, LW-FGAC is the only one providing a lightweight encryption approach. Also, we see that LW-FGAC is the only scheme that simultaneously meets all the features given in the table. We refer the reader to [37,38,39,40,41,42,43,44], to see more references related to attribute-based systems and wireless sensor networks.

## 3. System Architecture

In this section, we present the architecture of our proposed health system. We first describe the system model, and then we present the threat model of our system.

### 3.1. System Model

**Figure 2:**
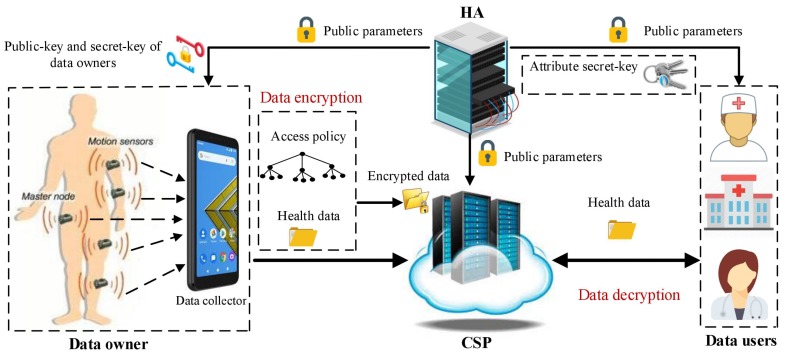
Architecture of our proposed LW-FGAC scheme.

As we have shown in Figure 2, our proposed system consists of four generic entities Healthcare Authority (HA), the Cloud Service Provider (CSP), several data owners, and several data users. In below, we describe the mentioned four entities:

- HA: This entity is responsible for initializing the health system and also generating secret-keys of data owners and data users according to their attributes.
- CSP: The CSP has almost unlimited computational and storage resources. Its primary responsibility is to provide storage and computational services. When data owners want to encrypt their collected data, they can outsource most of the computational operations of the encryption phase to the CSP. Moreover, data users can also use the CSP’s computational services. When a data user retrieves an encrypted health data, the CSP can help it to recover the associated data by performing most of the heavy computations of the decryption phase without learning any partial information about the underlying health data.
- Data owner: Data owners modeling the tiny wireless sensors attached to bodies of patients and employed to monitor the patients’ vital physiological parameters such as blood pressure, heart rate, diabetes, asthma, and etc. The health data collected by data owners first is encrypted under an access control policy and then transferred to a smart device. Finally, the health data are outsourced to the CSP for online/offline analyzing and long-term storage.
- Data owner: Data owners modeling smart devices that collect the health data from patients’ bodies and transfer the data to the CSP. The smart devices can be categorized into two following groups:
  1. Implanted and wearable sensors: These sensors usually embedded on the surface of a patient’s body or implanted in the deep tissue of a human body. Their main responsibility is to monitor the patients’ vital physiological parameters such as blood pressure, heart rate, diabetes, asthma, and etc. After collecting the health data, the sensors first partially encrypt the data under a predetermined access control policy. Then, the partially encrypted data are transferred to the data collector. Note that as the sensors usually have limited computational and power resources, the partial encryption process should be adequate sufficient and does not include costly operations.
  2. Data collector: A data collector could be the WBAN’s controller or a mobile device like a tablet or a smartphone. Its main responsibility is to transfer the collected partially encrypted health data to the CSP for completing the encryption process, long-term storage, and online/offline analyzing.
- Data user: Data users model health service providers such as hospitals, doctors, medical clinics, etc. They can be specified by a set of descriptive attributes. Each data user should obtain a secret-key corresponding to its attribute set. Its secret-key can decrypt an outsourced encrypted health data only if the attribute set associated with the secret-key satisfies the access control policy associated with the ciphertext.

**Figure 3:**
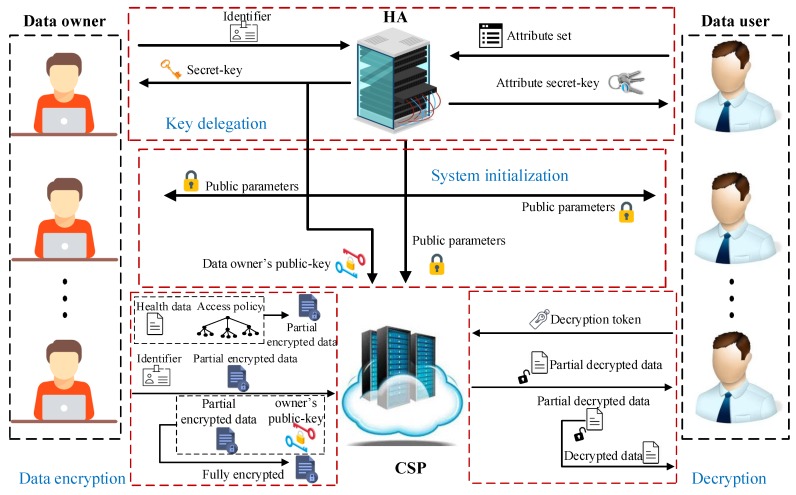
Workflow of our proposed LW-FGAC scheme.

In the following, we give an overview of our proposed LW-FGAC. As shown in Figure 3, our proposed scheme consists of four phases Systeminitialization, Key delegation, Data encryption, and Decryption described below:

- System initialization: This phase is managed by the HA. In this phase, the HA generates the public parameters and the master secret-key of the system. It publishes the public parameters to the other parties and keeps the master secret-key confidential by itself.
- Key delegation: This phase is operated by the HA. In this phase, public-key and secret-key of data owners as well as secret-keys of data users associated with their attributes are issued. Each data owner should ask the HA to generate its public-key and secret-key. The generated secret-key is given to the data owner, and the public-key is outsourced to the CSP. Also, in this phase, each data user possessing an attribute set can request its secret-key corresponding to the attribute set from the HA. The HA first checks if the data user has the attributes or not. If so, it provides the data user with an attribute secret-key.
- Data encryption: This phase is executed by data owners and the CSP. When a data owner wants to outsource its collected health data to the CSP, to provide confidentiality and access control, it should define an access control policy and encrypt the health data under it. However, as the computational power of the data owner (implanted and wearable sensors) is assumed to be limited, the heavy computational operations should be offloaded onto the CSP. Using its secret-key, the data owner (implanted and wearable sensors) first performs some lightweight computations and generates a partial ciphertext. Then, the data owner (data collector) gives the partially encrypted data to the CSP, and the CSP completes the encryption procedure. In this phase, the CSP cannot learn any partial information about the underlying health data.
- Decryption: This phase is managed by the CSP and data users. When a data user is authorized for accessing an outsourced health data, using its secret-key obtained in the key delegation phase, it can make a decryption query to the CSP. The CSP performs heavy operations associated with the decryption phase without obtaining any information about the data user’s secret-key and also the associated health data. Afterward, the data user can recover the health data by performing some lightweight computational operations.

### 3.2. Threat Model

The HA is assumed to be trustworthy. It does not collude with data users and does not gives unauthorized secret-keys to them. Data owners also are assumed to be trusted. They do not reveal the contents of their data to the other parties and do not grant access rights to unauthorized data users. The CSP is assumed to be honest but curious entity. It always executes the given protocols correctly, but it is curious to learn some unauthorized information about the outsourced health data. To gain some information about the outsourced data files, it may collude with unauthorized data users. Data users are assumed to be malicious. Although they do not reveal the contents of health data files if they are authorized to access them, they may try to learn some unauthorized information about the other outsourced health data through colluding with the CSP and the other data users.

## 4. Preliminaries

For an arbitrary set *S*, let x←S denote the random selection of an element x∈S. Also, for algorithm A, let O←A(I) denote executing A on input *I* and outputting *O*. In the following, we present some related cryptographic notions.

### 4.1. Cryptographic Background

Bilinear map: Consider two cyclic groups $G_{1}$ and $G_{2}$ of a prime order *q*. A function $\hat{e}:G_{1}\times G_{1}\to G_{2}$ is said to be a bilinear map if the following conditions hold:

- Bilinearity: $\hat{e}\left(g^{a},g^{b}\right)=\hat{e}\left(g^{b},g^{a}\right)=\hat{e}{\left(g,g\right)}^{ab}$, For each $a,b\in Z_{q}$ and $g\in G_{1}$,
- Non-degeneracy: There is a $g\in G_{1}$ such that $\hat{e}\left(g,g\right)\neq 1$.
- Computability: There exists an efficient algorithm computing $\hat{e}\left(g,h\right)$, for any $g,h\in G_{1}$.

Assume that G is a probabilistic polynomial-time (PPT) algorithm that $\left(\lambda ,q,G_{1},G_{2},\hat{e}\right)\leftarrow G\left(1^{\lambda }\right)$, where λ is the security parameter of the system and $\left(q,G_{1},G_{2},\hat{e}\right)$ is the same as before. In this work, we consider the following assumption called decisional bilinear Diffie Hellman (DBDH) on G:

Decisional Bilinear Diffie Hellman assumption (DBDH): Consider $\left(\lambda ,q,G_{1},G_{2},\hat{e}\right)\leftarrow G\left(1^{\lambda }\right)$, $g\leftarrow G_{1}$ and $\alpha ,\beta ,\gamma \leftarrow Z_{q}$. The DBDH assumption states that for all PPT adversaries A there is a negligible function negl such that

(1) $$|\mathrm{Pr}\left(A\left(\lambda ,q,g,g^{\alpha },g^{\beta },g^{\gamma },g^{\alpha \beta \gamma },G_{1},G_{2},\hat{e}\right)=1\right)-\mathrm{Pr}\left(A\left(\lambda ,q,g,g^{\alpha },g^{\beta },g^{\gamma },g^{z},G_{1},G_{2},\hat{e}\right)=1\right)|\leq negl\left(\lambda \right),$$

where the above probabilities are taken over the random selection of g∈G and $\alpha ,\beta ,\gamma ,z\in Z_{q}$, and also the randomness employed in G and A.

### 4.2. Access Trees

In an access tree, each leaf is associated with a unique attribute, and each inner node represents a threshold value. Also, the threshold value of each leaf node is assumed to be 1. Suppose that T is an access tree, $v_{a}$ is the leaf associated with an attribute *a*, $k_{v}$ is the threshold value associated with a node *v* in T, $R_{T}$ is the root node of T, $L_{T}$ is the leaf node set of T, and $T_{v}$ is a subtree of T rooted at a node *v*.

Let U be the universal attribute set, and T be an access tree on U. For a given attribute set Att⊆U and a node *v* in T, let $F_{T_{v}}$ be a function mapping Att to {0,1} and performing as follows:

- When *v* is a leaf node corresponding to an attribute *a*, $F_{T_{v}}\left(Att\right)=1$ if a∈Att, and 0 otherwise.
- When *v* is an inner node, $F_{T_{v}}\left(Att\right)=1$ if and only if *v* has at least $k_{v}$ children $c_{1},\ldots ,c_{k_{v}}$ that $F_{T_{c_{i}}}\left(Att\right)=1$, for any $i=1,\ldots k_{v}$.

We say that an attribute set Att satisfies an access tree T if $F_{T_{R_{T}}}\left(Att\right)=1$.

Suppose that *q* is a prime number, and T is an access tree. Consider an algorithm ${\left\{q_{v}\left(0\right)\right\}}_{v\in L_{T}}\leftarrow {Share}_{q}\left(T,r\right)$ which shares a secret $r\in Z_{q}$ according to T and *q* and performs as below:

- It generates a $\left(k_{R_{T}}-1\right)$-degree polynomial $q_{R_{T}}$ for $R_{T}$ such that $q_{R_{T}}\left(0\right)=r$, and its other coefficients are chosen uniformly at random from $Z_{q}$.
- For each node *v* having a polynomial $q_{v}$, it generates a polynomial $q_{c_{i}}$ for the *i*-th child of *v* such that $q_{c_{i}}\left(0\right)=q_{v}\left(i\right)$, and the other its coefficients are uniform elements of $Z_{q}$.

When this algorithm stops, it assigns a value $q_{v}\left(0\right)$ to each leaf node *v* in the tree.

## 5. System Definition and Security Model

In this section we present the system definition and the secrity model. Table 2 presents the notations used in this section.

### 5.1. Definition of LW-FGAC

LW-FGAC scheme is a tuple of PPT algorithms (Setup,User.KeyGen,Owner.KeyGen,Part.Enc,Full.Enc,TokenGen,Part.Dec,Full.Dec) defined as below:

- Setup(λ,U): This algorithm is operated by the HA. It takes as input the security parameter λ and the universal attribute set U. It outputs public parameters params and the master secret-key MSK.
- $User.KeyGen(params,MSK,id_{u},Att_{u})$: This algorithm is executed by the CSP. On input the public parameters params, the master secret-key MSK, a data user’s identifier $id_{u}$, and an attribute set $Att_{u}$, this algorithm outputs a secret-key $SK_{u}$ associated with $id_{u}$ and $Att_{u}$.
- Owner.KeyGen(params): This algorithm can be run by a data owner or the HA. It inputs the public parameters params and outputs a pair of secret-key and public-key $\left(SK_{O},PK_{O}\right)$.
- $Part.Enc(params,T,SK_{O},M)$: A data owner executes this algorithm. The public parameters of the system, an access tree T, the data owner’s secret-key, and a message *M* are the input of the algorithm. This algorithm outputs a partial ciphertext $PCT_{T}$ associated with the message *M* and the access tree T.
- $Full.Enc(params,PCT_{T},PK_{O})$: The CSP runs this algorithm. This algorithm takes the public parameters params, a partial ciphertext $PCT_{T}$, and a data owner’s public-key $PK_{O}$. It outputs a ciphertext $CT_{T}$.
- $TokenGen(params,id_{u},SK_{u},CT_{T})$: This algorithm is executed by a data user. On input the pubic parameters params, a data user’s identifier $id_{u}$, a secret-key $SK_{u}$, and a ciphertext $CT_{T}$, this algorithm returns a private-key *k* and a decryption token $TK_{u}$, or it outputs an error message ⊥.
- $Part.Dec(params,CT_{T},TK_{u})$: The CSP runs this algorithm. It takes as input the public parameters params, a ciphertext $CT_{T}$, and a decryption token $TK_{u}$. This algorithm outputs a partial decrypted ciphertext $M^{\prime }$.
- $Full.Dec(params,M^{\prime },k)$: A data user operates this algorithm. On input the public parameters params, the partial decrypted ciphertext $M^{\prime }$, and its associated private-key *k*, this algorithm returns the message associated with $M^{\prime }$.

**Definition** **1.** *We say that an LW−FGAC scheme* Π *is correct if for any security parameter λ, universal attribute set U, public parameters and master secret-key (params,MSK)←Setup(λ,U), attribute set $Att_{u}$, identifier $id_{u}$, access tree T satisfied by $Att_{u}$, secret-key $SK_{u}\leftarrow User.KeyGen\left(params,MSK,id_{u},Att_{u}\right)$, public-key and secret-key $\left(SK_{O},PK_{O}\right)\leftarrow Owner.KeyGen\left(params\right)$, message M, partial ciphertext $PCT_{T}\leftarrow Part.Enc\left(params,T,SK_{O},M\right)$, and ciphertext $CT_{T}\leftarrow Full.Enc\left(params,PCT_{T},PK_{O}\right)$, we have:*

(2) $$Full.Dec(params,M^{\prime },k)=M,$$

*where $M^{\prime }\leftarrow Part.Dec\left(params,CT_{T},TK_{u}\right)$ and $TK_{u}\leftarrow TokenGen\left(params,id_{u},SK_{u},CT_{T}\right)$.*

### 5.2. Security Definition

Security of LW-FGAC requires that for any PPT adversary modeling the CSP colluding with unauthorized data users, the advantage of the adversary in learning partial information about encrypted data files is a negligible function in the security parameter of the system. In other words, the adversary is unable to distinguish the encryption of two data files of its choice. We formalize the security requirement by using the following indistinguishability experiment.

Indistinguishability experiment ${LW-FGAC}_{A,\Pi }\left(\lambda \right)$:

Let Π=(Setup,User.KeyGen,Owner.KeyGen,Part.Enc,Full.Enc,TokenGen,Part.Dec,Full.Dec) be an LW-FGAC scheme and A be a PPT adversary. Consider the following experiment:

1. Setup: A challenger chooses a security parameter λ and a universal attribute set U. It executes (params,MSK)← Setup (λ,U). params is given to A and MSK is maintained by the challenger.
2. Phase 1: For polynomially many times, A makes some queries to the following oracle, and for each data user with identifier $id_{u}$, the challenger maintains a list $L_{id_{u}}$ which is initially empty.
  $O_{User.KeyGen}\left(Att,id_{u}\right)$: The challenger runs $SK_{u}\leftarrow UKeyGen\left(PK,MSK,Att,id_{u}\right)$ and returns $SK_{u}$ to the adversary. It also substitutes $L_{id_{u}}\cup Att$ with $L_{id_{u}}$.
3. Challenge: A declares an access tree $T^{*}$ and two equal-length messages $M_{0}$ and $M_{1}$. The challenger checks if there is an identifier $id_{u}$ such that $L_{id_{u}}$ satisfies $T^{*}$ or not. If so, the challenger stops and returns 0. Otherwise, it first selects b←{0,1} and an identifier $id_{O}$. Then, it runs $\left(SK_{O},PK_{O}\right)\leftarrow Owner.KeyGen\left(params\right)$ and $PCT_{T}^{b}\leftarrow Part.Enc\left(params,T,SK_{O},M_{b}\right)$. $PK_{O}$ and $PCT_{T}^{b}$ are given to A.
4. Phase 2: A makes more queries to the oracle $O_{\mathrm{User}.\mathrm{KeyGen}}\left(Att,id_{u}\right)$ and the challenger answers it provided $Att\cup L_{id_{u}}$ does not satisfy $T^{*}$.
5. Guess: A outputs a bit $b^{\prime }\in \left\{0,1\right\}$.

The output of the experiment is defined to be 1 if $b=b^{\prime }$, and 0 otherwise. We say that the adversary A wins the game, and we write ${LW-FGAC}_{A,\Pi }\left(\lambda \right)=1$ if the experiment’s output is equal to 1.

**Definition** **2.** *An LW−FGAC scheme* Π *is said to be secure if for all PPT adversaries A there exists a negligible function negl such that*

(3) $$Pr\left({LW-FGAC}_{A,\Pi }\left(\lambda \right)=1\right)\leq \frac{1}{2}+negl\left(\lambda \right).$$

## 6. Our Construction

In this section, we present our proposed LW-FGAC scheme. As mentioned in Section 3.1, our proposed scheme consists of four phases System initialization, Key delegation, Data encryption, and Decryption. In the following, the mentioned four phases are described in detail. The notations employed in our construction are given in Table 2.

### 6.1. System Initialization

In this phase, the HA selects a security parameter λ and a universal attribute set U. Then, it executes (params,MSK)←Setup(λ,U) as follows and publishes params to the other entities.

Setup(λ,U): This algorithm runs $\left(\lambda ,q,G_{1},G_{2},\hat{e}\right)\leftarrow G\left(1^{\lambda }\right)$ and selects $P_{0},P_{1},P_{2},X_{1}\leftarrow G_{1}$, and $x_{0}\leftarrow Z_{q}$. Then, for each i∈U, it chooses $sk_{i}\leftarrow Z_{q}$ and computes $PK_{i}=sk_{i}P_{0}$. It sets

(4) $$MSK=(x_{0},P_{1},X_{1},{\left\{sk_{i}\right\}}_{i=1}^{m})$$

and

(5) $$params=(\lambda ,G_{1},G_{2},\hat{e},P_{0},P_{2},E_{1},E_{2},{\left\{PK_{i}\right\}}_{i=1}^{m}),$$

as the master secret-key and the global public parameters of the system, respectively, where $E_{1}=\hat{e}\left(x_{0}P_{0},P_{1}\right)$ and $E_{2}=\hat{e}\left(P_{0},X_{1}\right)$.

### 6.2. Key Delegation

**Figure 4:**
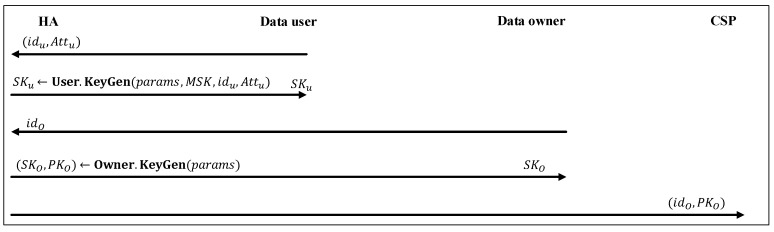
Key delegation phase.

As shown in Figure 4, in this phase, the HA provides data users with some secret-keys according to their attributes and also provides each data owner with a pair of public-key and secret-key. Each data user possessing an attribute set $Att_{u}$ should first select a unique identifier $id_{u}$ and ask the HA to generate its secret-key. The HA runs $SK_{u}\leftarrow User.KeyGen\left(params,MSK,id_{u},Att_{u}\right)$ and returns $SK_{u}$ to the data user. Also, each data owner with identifier $id_{O}$ can request its public-key and secret-key from the HA. The HA runs $\left(SK_{O},PK_{O}\right)\leftarrow Owner.KeyGen\left(params\right)$ and returns $SK_{O}$ to the data owner. $\left(id_{O},PK_{O}\right)$ is also outsourced to the CSP. Note that secret-key and public-key of a data owner can be generated by itself. However, as its computational power is assumed to be limited, this task usually is outsourced to the HA. In the following, we describe the mentioned two algorithms:

$User.KeyGen(params,MSK,id_{u},Att_{u})$: It calculates:

(6) $$SK_{i,u}=x_{0}P_{1}+X_{1}+sk_{i}id_{u},$$

for each $i\in Att_{u}$, and outputs $SK_{u}={\left\{SK_{i,u}\right\}}_{i\in Att_{u}}$.

Owner.KeyGen(params): It selects $d_{O}\leftarrow Z_{q}$ and calculates $PK_{O}^{\left(1\right)}=E_{2}^{d_{O}}$, $PK_{O}^{\left(2\right)}=d_{O}P_{0}$, $PK_{O}^{\left(3\right)}=d_{O}P_{2}$ and $PK_{i,O}=d_{O}\left(PK_{i}-P_{2}\right)$, for each i∈U. It returns $\left(SK_{O},PK_{O}\right)$, where $SK_{O}=d_{O}$ and $PK_{O}=\left(PK_{O}^{\left(1\right)},PK_{O}^{\left(2\right)},PK_{O}^{\left(3\right)},{\left\{PK_{i,O}\right\}}_{i\in U}\right)$.

### 6.3. Data Encryption

**Figure 5:**
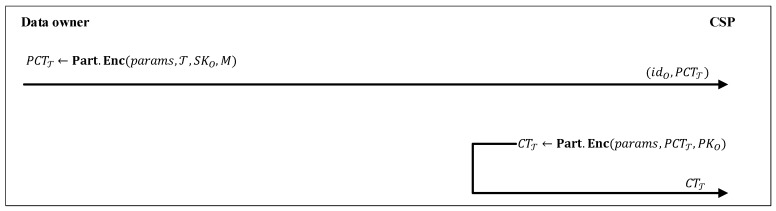
Data encryption phase.

As shown in Figure 5, in this phase, data owners encrypt their data by outsourcing most of the computational operations to the CSP. A data owner with identifier $id_{O}$ and public-key and secret-key $\left(SK_{O},PK_{O}\right)$ that wants to encrypt a message *M* defines an access tree T and runs $PCT_{T}\leftarrow Part.Enc\left(params,T,SK_{O},M\right)$ to generate a partial ciphertext $PCT_{T}$. The data owner makes a request $\left(id_{O},PCT_{T}\right)$ to the CSP to complete the encryption procedure. Then, the CSP executes $CT_{T}\leftarrow Full.Enc\left(params,PSK_{T},PK_{O}\right)$ and generates a ciphertext associated with the message *M* and the access tree T. The mentioned two algorithms are presented below:

$Part.Enc(params,T,SK_{O},M)$: It selects $r\leftarrow Z_{q}$ and runs ${\left\{q_{v_{i}}\left(0\right)\right\}}_{v_{i}\in L_{T}}\leftarrow {Share}_{q}\left(r+SK_{O},T\right)$. Then, it calculates $C_{1}=E_{1}^{-r}M$, $\tilde{r}=r+SK_{O}$ and returns partial ciphertext $PCT_{T}=\left(T,C_{1},\tilde{r},{\left\{q_{v_{i}}\left(0\right)\right\}}_{v_{i}\in L_{T}}\right)$.

$Full.Enc(params,PCT_{T},PK_{O})$: Given a partial ciphertext $PCT_{T}=\left(T,C_{1},\tilde{r},{\left\{q_{v_{i}}\left(0\right)\right\}}_{v_{i}\in L_{T}}\right)$ and a data owner’s public-key $PK_{O}=\left(PK_{O}^{\left(1\right)},PK_{O}^{\left(2\right)},PK_{O}^{\left(3\right)},{\left\{PK_{i,O}\right\}}_{i\in U}\right)$, it calculates

(7) $$C_{2}=\tilde{r}P_{2}-PK_{O}^{\left(3\right)}=rP_{2},$$

(8) $$C_{3}=E_{2}^{-\tilde{r}}\left(PK_{O}^{\left(1\right)}\right)=E_{2}^{-r},$$

and for any leaf node $v_{i}$ in T, it sets

(9) $$C_{v_{i}}^{\left(1\right)}=q_{v_{i}}\left(0\right)P_{0}-PK_{O}^{\left(2\right)}=\left(q_{v_{i}}\left(0\right)-SK_{O}\right)P_{0},$$

(10) $$C_{v_{i}}^{\left(2\right)}=q_{v_{i}}\left(0\right)\left(PK_{i}-P_{2}\right)-PK_{i,O}=\left(q_{v_{i}}\left(0\right)-SK_{O}\right)\left(PK_{i}-P_{2}\right).$$

Finally, this algorithm outputs a ciphertext

(11) $$CT_{T}=\left(T,C_{1},C_{2},C_{3},{\left\{C_{v_{i}}^{\left(1\right)}\right\}}_{v_{i}\in L_{T}},{\left\{C_{v_{i}}^{\left(2\right)}\right\}}_{v_{i}\in L_{T}}\right).$$

### 6.4. Decryption

**Figure 6:**
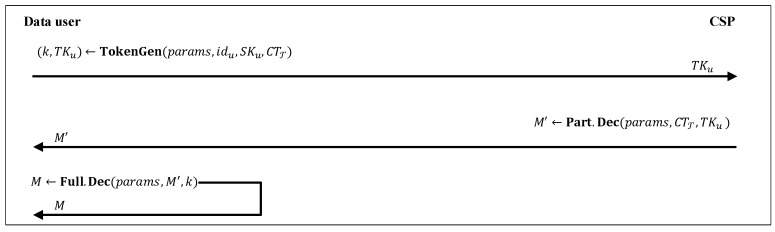
Decryption phase.

As we have shown in Figure 6, in this phase, by outsourcing the heavy computational operations to the CSP, a data user can recover its desired data. Assume that $CT_{T}$ has been retrieved from the CSP. To decrypt the ciphertext, a data user with secret-key $SK_{u}$ and identifier $id_{u}$ first executes $TK_{u}\leftarrow TokenGen\left(params,id_{u},SK_{u},CT_{T}\right)$ and generates a decryption token $TK_{u}$. It sends a decryption request $\left(CT_{T},TK_{u}\right)$ to the CSP. Then, the CSP runs $M^{\prime }\leftarrow Part.Dec\left(params,CT_{T},TK_{u}\right)$ and returns the partial decrypted ciphertext $M^{\prime }$ to the data user. The data user can run the lightweight algorithm $M\leftarrow Full.Dec(parms,M^{\prime },k)$ and recover the associated message *M*. Detail of the mentioned three algorithms are given below:

$TokenGen(params,id_{u},SK_{u},CT_{T})$: Given a data user’s secret-key $SK_{u}={\left\{SK_{i,u}\right\}}_{i\in Att_{u}}$ associated with an attribute set $Att_{u}$, a ciphertext $CT_{T}$ associated with an access tree T, and an identifier $id_{u}$, this algorithm checks if there is an attribute set $S\subseteq Att_{u}$ satisfying T or not. If not, it returns ⊥. Otherwise, it selects $k\leftarrow Z_{q}$ and calculates $K=k\;id_{u}$ and $K_{i}=k\;SK_{i,u}$, for each i∈S. It outputs a private-key *k* and a token $TK_{u}=\left(K,{\left\{K_{i}\right\}}_{i\in S}\right)$.

$Part.Dec(params,CT_{T},TK_{u})$: Given a ciphertext $CT_{T}=\left(T,{\left\{C_{i}\right\}}_{i=1}^{4},{\left\{C_{v_{i}}^{\left(1\right)}\right\}}_{v_{i}\in L_{T}},{\left\{C_{v_{i}}^{\left(2\right)}\right\}}_{v_{i}\in L_{T}}\right)$ and a token $TK_{u}=\left(K,{\left\{K_{i}\right\}}_{i\in S}\right)$, it first computes

(12) $$\begin{array}{cc} L_{i} & =\frac{\hat{e}\left(K_{i},C_{v_{i}}^{\left(1\right)}\right)}{\hat{e}\left(K,-C_{v_{i}}^{\left(2\right)}\right)}\\ \; & =E_{1}^{kq_{v_{i}}\left(0\right)}E_{2}^{kq_{v_{i}}\left(0\right)}\hat{e}{\left(id_{u},P_{2}\right)}^{kq_{v_{i}}\left(0\right)}, \end{array}$$

for each i∈S. Then, by using the polynomial interpolation method, it computes

(13) $$L=E_{1}^{kr}E_{2}^{kr}\hat{e}{\left(id_{u},P_{2}\right)}^{kr}.$$

Finally, it returns $M^{\prime }=\left(C^{\prime },C_{1}\right)$, where

(14) $$C^{\prime }=\frac{L}{\hat{e}\left(K,C_{2}\right)}=E_{1}^{kr}E_{2}^{kr}.$$

$Full.Dec(parms,M^{\prime },k)$: On input a partial decrypted ciphertext $M^{\prime }$ and its associated private-key *k*, this algorithm outputs a message

(15) $$M=C^{\prime k^{-1}}C_{1}C_{3}.$$

## 7. Correctness and Security Analysis

In this section, we first show that our proposed scheme is correct. Then, we prove its security in the standard model.

### 7.1. Correctness Proof

**Theorem** **1.** *Our proposed LW-FGAC scheme is correct.*

**Proof.**  We prove that LW-FGAC fulfills Definition 1. Given (params,MSK)←Setup(λ,U), an attribute set $Att_{u}$, an identifier $id_{u}$, an access tree T satisfied by $Att_{u}$, a message *M*, $SK_{u}\leftarrow User.KeyGen\left(params,MSK,id_{u},Att_{u}\right)$, $\left(SK_{O},PK_{O}\right)\leftarrow Owner.KeyGen\left(params\right)$, $PCT_{T}\leftarrow Part.Enc\left(params,T,SK_{O},M\right)$, $CT_{T}\leftarrow Full.Enc\left(params,PCT_{T},PK_{O}\right)$, we show that the output of the decryption phase is equal to *M*. Let $CT_{T}=\left(T,{\left\{C_{i}\right\}}_{i=1}^{4},{\left\{C_{v_{i}}^{\left(1\right)}\right\}}_{v_{i}\in L_{T}},{\left\{C_{v_{i}}^{\left(2\right)}\right\}}_{v_{i}\in L_{T}}\right)$, and $TK_{u}=\left(K,{\left\{K_{i}\right\}}_{i\in S}\right)$ be a decryption token generated by $TokenGen(params,id_{u},SK_{u},CT_{T})$, where $S\subset Att_{u}$ satisfies T. We first prove the correctness of Equation (12). We have:

(16) $$\begin{array}{cc} L_{i} & =\frac{\hat{e}\left(K_{i},C_{v_{i}}^{\left(1\right)}\right)}{\hat{e}\left(K,-C_{v_{i}}^{\left(2\right)}\right)}\\ \; & =\frac{\hat{e}\left(kSK_{i,u},q_{v_{i}}\left(0\right)P_{0}\right)}{\hat{e}\left(kid_{u},q_{v_{i}}\left(0\right)\left(-P_{2}+PK_{i}\right)\right)}\\ \; & =\frac{\hat{e}\left(kx_{0}P_{1}+kX_{1}+ksk_{i}id_{u},q_{v_{i}}\left(0\right)P_{0}\right)}{\hat{e}{\left(kid_{u},P_{2}\right)}^{-q_{v_{i}}\left(0\right)}\hat{e}{\left(kid_{u},PK_{i}\right)}^{q_{v_{i}}\left(0\right)}}\\ \; & =\frac{\hat{e}\left(kx_{0}P_{1},q_{v_{i}}\left(0\right)P_{0}\right)\hat{e}\left(kX_{1},q_{v_{i}}\left(0\right)P_{0}\right)\hat{e}{\left(kid_{u},PK_{i}\right)}^{q_{v_{i}}\left(0\right)}}{\hat{e}{\left(kid_{u},P_{2}\right)}^{-q_{v_{i}}\left(0\right)}\hat{e}{\left(kid_{u},PK_{i}\right)}^{q_{v_{i}}\left(0\right)}}\\ \; & =\hat{e}\left(kx_{0}P_{1},q_{v_{i}}\left(0\right)P_{0}\right)\hat{e}\left(kX_{1},q_{v_{i}}\left(0\right)P_{0}\right)\hat{e}{\left(kid_{u},P_{2}\right)}^{q_{v_{i}}\left(0\right)}\\ \; & =\hat{e}{\left(x_{0}P_{1},P_{0}\right)}^{kq_{v_{i}}\left(0\right)}\hat{e}{\left(X_{1},P_{0}\right)}^{kq_{v_{i}}\left(0\right)}\hat{e}{\left(id_{u},P_{2}\right)}^{kq_{v_{i}}\left(0\right)}\\ \; & =E_{1}^{kq_{v_{i}}\left(0\right)}E_{2}^{kq_{v_{i}}\left(0\right)}\hat{e}{\left(id_{u},P_{2}\right)}^{kq_{v_{i}}\left(0\right)}. \end{array}$$

So, Equation (12) is correct. Also, the correctness of Equations (13) and (14) is clear. Moreover, we see that

(17) $$\begin{array}{cc} C^{\prime k^{-1}}C_{1}C_{3} & ={\left(E_{1}^{kr}E_{2}^{kr}\right)}^{k^{-1}}ME_{1}^{-r}E_{2}^{-r}\\ \; & =\left(E_{1}^{r}E_{2}^{r}\right)ME_{1}^{-r}E_{2}^{-r}\\ \; & =M. \end{array}$$

It proves the theorem. □

### 7.2. Security Proof

**Theorem** **2.** *If the DBDH problem is hard relative to G, then LW-FGAC construction is secure in the standard model.*

**Proof.**  Let Π be our proposed LW-FGAC scheme, and A is a PPT adversary in the experiment ${LW-FGAC}_{A,\Pi }\left(n\right)=1$ introduced in Section 6. In the following, we show that there exists a negligible function negl such that:

(18) $$\mathrm{Pr}\left({LW-FGAC}_{A,\Pi }\left(\lambda \right)=1\right)\leq \frac{1}{2}+negl\left(\lambda \right),$$

where λ is the security parameter of the system. Suppose that $A^{\prime }$ is another PPT adversary that attempts to solve the DBDH problem. Recall that the adversary $A^{\prime }$ receives $\left(\lambda ,q,G_{1},G_{2},\hat{e},P,\alpha P,\beta P,\gamma P,\hat{e}{\left(P,P\right)}^{z}\right)$, where $P\leftarrow G_{1}$, $\alpha ,\beta ,\gamma \leftarrow Z_{q}$, and z is equal to αβγ or is a uniform element of $Z_{q}$. The aim of $A^{\prime }$ is to determine the case of z. $A^{\prime }$ runs A as a subroutine as follows:

1. Setup: At first, $A^{\prime }$ considers a universal attribute set U, and for each i∈U, chooses a uniform element $sk_{i}\in Z_{q}$. Then, it selects $t\leftarrow Z_{q}$ and $X\leftarrow G_{1}$ and sets
  (19) $$P_{0}=P,$$
  (20) $$P_{1}=\alpha P,$$
  (21) $$P_{2}=tP,$$
  (22) $$x_{0}P_{0}=\beta P,$$
  (23) $$E_{1}=\hat{e}\left(\beta P,P_{1}\right),$$
  (24) $$E_{2}=\hat{e}\left(P,X\right).\hat{e}{\left(\alpha P,\beta P\right)}^{-1},$$
  and
  (25) $$PK_{i}=sk_{i}P,$$
  for any attribute i∈U. $A^{\prime }$ gives $params=(\lambda ,q,G_{1},G_{2},\hat{e},P_{0},P_{1},P_{2},E_{1},E_{2},,{\left\{PK_{i}\right\}}_{i\in U})$ to A as the global public parameters of the system. Note that, if we assume that the master secret-key $MSK=(x_{0},P_{1},X_{1},{\left\{sk_{a_{i}}\right\}}_{i=1}^{m})$ is chosen such that the following equations
  (26) $$x_{0}=\beta ,$$
  (27) $$\begin{array}{cc} X & =\beta P_{1}+X_{1}=\alpha \beta P+X_{1}, \end{array}$$
  hold, then one can see that
  (28) $$\begin{array}{cc} E_{2} & =\hat{e}\left(P,X\right).\hat{e}{\left(\alpha P,\beta P\right)}^{-1}\\ \; & =\hat{e}\left(P,\alpha \beta P+X_{1}\right).\hat{e}{\left(\alpha P,\beta P\right)}^{-1}\\ \; & =\hat{e}\left(P,\alpha \beta P\right).\hat{e}\left(P,X_{1}\right).\hat{e}{\left(\alpha P,\beta P\right)}^{-1}\\ \; & =\hat{e}\left(\alpha P,\beta P\right).\hat{e}\left(P,X_{1}\right).\hat{e}{\left(\alpha P,\beta P\right)}^{-1}\\ \; & =\hat{e}\left(P,X_{1}\right)\\ \; & \overset{\left(19\right)}{=}\hat{e}\left(P_{0},X_{1}\right). \end{array}$$
  So, $E_{2}$ is chosen correctly. The correctness of the other components of params can be easily checked.
2. Phase 1: For any data user with identifier $id_{u}$, $A^{\prime }$ makes a list $L_{id_{u}}$ which is initially empty. When A submits a query $O_{\mathrm{User}.\mathrm{KeyGen}}\left(Att,id_{u}\right)$, it sets $L_{id_{u}}=L_{id_{u}}\cup Att$ and computes
  (29) $$SK_{i,u}=X+sk_{i}id_{u}.$$
  Combining Equations (20) and (22), we have:
  (30) $$\begin{array}{cc} SK_{i,u} & =X+sk_{i}id_{u}\\ \; & =\alpha \beta P+X_{1}+sk_{i}id_{u}\\ \; & =\beta \left(\alpha P\right)+X_{1}+sk_{i}id_{u}\\ \; & =\beta P_{1}+X_{1}+sk_{i}id_{u}\\ \; & =x_{0}P_{1}+X_{1}+sk_{i}id_{u}. \end{array}$$
  Also, by Equations (6) and (30), we see that $SK_{i,u}$ in Equation (29) is a valid secret-key.
3. Challenge: A declares an access tree $T^{*}$ and two equal-length messages $M_{0}$ and $M_{1}$ such that there is no data user with identifier $id_{u}$ such that $L_{id_{u}}$ satisfies $T^{*}$. $A^{\prime }$ selects b←{0,1} and $r^{\prime }\leftarrow Z_{q}$ and assumes that for an unknown $SK_{O}\in Z_{q}$, $r^{\prime }=\gamma +SK_{O}$. It sets
  (31) $$PK_{O}^{\left(1\right)}=E_{2}^{r^{\prime }}\hat{e}\left(-\gamma P,X\right)\hat{e}{\left(P,P\right)}^{z},$$
  (32) $$PK_{O}^{\left(2\right)}=r^{\prime }P-\gamma P,$$
  (33) $$PK_{O}^{\left(3\right)}=r^{\prime }P_{2}-t\gamma P,$$
  and for each i∈U, it calculates
  (34) $$PK_{i,O}=r^{\prime }\left(PK_{i}-P_{2}\right)-\left(sk_{i}\gamma P-t\gamma P\right).$$
  Then, it runs ${\left\{q_{v_{i}}\left(0\right)\right\}}_{v_{i}\in L_{T^{*}}}\leftarrow Share\left(r^{\prime },q,T^{*}\right)$ and calculates
  (35) $$C_{1}=\hat{e}{\left(P,P\right)}^{-z}M_{b}.$$
  Afterward, it sets $PCT_{T^{*}}^{b}=\left(T^{*},C_{1},{\left\{q_{v_{i}}\left(0\right)\right\}}_{v_{i}\in L_{T^{*}}}\right)$. Finally, it returns $PCT_{T^{*}}^{b}$ and $PK_{O}=\left(PK_{O}^{\left(1\right)},PK_{O}^{\left(2\right)},PK_{O}^{\left(3\right)},{\left\{PK_{i,O}\right\}}_{i\in U}\right)$ to A. We see that
  $$\begin{array}{cc} PK_{O}^{\left(2\right)} & =r^{\prime }P-\gamma P\\ \; & =(\gamma +SK_{O})P-\gamma P\\ \; & =SK_{O}P\\ \; & =SK_{O}P_{0}, \end{array}$$
  $$\begin{array}{cc} PK_{O}^{\left(2\right)} & =r^{\prime }P_{2}-t\gamma P\\ \; & =(\gamma +SK_{O})tP-t\gamma P\\ \; & =SK_{O}tP\\ \; & =SK_{O}P_{2}, \end{array}$$
  and
  (36) $$\begin{array}{cc} PK_{i,O} & =r^{\prime }\left(PK_{i}-P_{2}\right)-\left(sk_{i}\gamma P-t\gamma P\right)\\ \; & =\left(SK_{O}+\gamma \right)\left(PK_{i}-P_{2}\right)-\gamma \left(PK_{i}-P_{2}\right)\\ \; & =SK_{O}\left(PK_{i}-P_{2}\right). \end{array}$$
  Therefore, $PK_{O}^{\left(2\right)}$, $PK_{O}^{\left(3\right)}$, and $PK_{i,O}$, for each i∈U, are chosen correctly. Also, when z=αβγ,
  (37) $$\begin{array}{cc} PK_{O}^{\left(1\right)} & =E_{2}^{r^{\prime }}\hat{e}\left(-\gamma P,X\right)\hat{e}{\left(P,P\right)}^{z}\\ \; & =E_{2}^{r^{\prime }}\hat{e}\left(-\gamma P,X\right)\hat{e}{\left(P,P\right)}^{\alpha \beta \gamma }\\ \; & =E_{2}^{r^{\prime }}\hat{e}{\left(P,X\right)}^{-\gamma }\hat{e}{\left(\alpha P,\beta P\right)}^{\gamma }\\ \; & \overset{\left(28\right)}{=}E_{2}^{r^{\prime }}E_{2}^{-\gamma }\\ \; & \;=E_{2}^{SK_{O}}, \end{array}$$
  and
  (38) $$\begin{array}{cc} C_{1} & =\hat{e}{\left(P,P\right)}^{-z}M_{b}\\ \; & =\hat{e}{\left(\alpha P,\beta P\right)}^{-\gamma }M_{b}\\ \; & =E_{1}^{-\gamma }M_{b}. \end{array}$$
  Thus, assuming z=αβγ and the random element r in Part.Enc algorithm described in Section 6.3 is equal to γ, one can see that $PK_{O}$ and $PCT_{T^{*}}^{b}$ are chosen correctly.
4. Phase 2: A makes more queries for data users’ secret-keys with the same restriction mentioned in the experiment presented in Section 5.2, and the adversary $A^{\prime }$ responds to the queries similar to Phase 1.
5. The adversary A outputs a bit $b^{\prime }\in \left\{0,1\right\}$.

Once the adversary $A^{\prime }$ receives $b^{\prime }$, it checks whether $b=b^{\prime }$ or not. If so, it outputs 1. Otherwise, it returns 0.

As we have seen, if z=αβγ, then $PK_{O}$ and $PCT_{T^{*}}^{b}$ are valid and therefore

(39) $$\begin{array}{c} \mathrm{Pr}\left(A^{\prime }\left(\lambda ,q,P,G_{1},G_{2},\hat{e},\alpha P,\beta P,\gamma P,\hat{e}{\left(P,P\right)}^{\alpha \beta \gamma }\right)=1\right)=\mathrm{Pr}\left({LW-FGAC}_{A,\Pi }\left(\lambda \right)=1\right). \end{array}$$

Also, it is clear that, if $z\in Z_{q}$ is a uniform element, then the adversary A cannot get any partial information about $M_{b}$. Thus,

(40) $$\begin{array}{c} \mathrm{Pr}\left(A^{\prime }\left(\lambda ,q,PG_{1},G_{2},\hat{e},\alpha P,\beta P,\gamma P,\hat{e}{\left(P,P\right)}^{z}\right)=1\right)=\frac{1}{2}. \end{array}$$

On the other hand, by the hardness assumption of the DBDH problem, we have

(41) $$\begin{array}{cc} | & \mathrm{Pr}(A^{\prime }\left(\lambda ,q,P,G_{1},G_{2},\hat{e},\alpha P,\beta P,\gamma P,D=\hat{e}{\left(P,P\right)}^{\alpha \beta \gamma }\right)=1)-\\ & \mathrm{Pr}(A^{\prime }\left(\lambda ,q,PG_{1},G_{2},\hat{e},\alpha P,\beta P,\gamma P,D=\hat{e}{\left(P,P\right)}^{z}\right)=1)|\leq negl\left(\lambda \right), \end{array}$$

for a negligible function negl. Combining Equations (39), (40), and (41), we get

(42) $$\begin{array}{c} \mathrm{Pr}\left({LW-FGAC}_{A,\Pi }\left(\lambda \right)=1\right)\leq \frac{1}{2}+negl\left(\lambda \right). \end{array}$$

This proves the theorem. □

**Corollary** **1.** *Our proposed system provides a secure lightweight encryption mechanism.*

**Proof.**  As we have seen in Theorem 1, the ciphertext generated by the lightweight encryption process is valid and can be decrypted by the algorithms presented in Section 6.4. Also, considering the security game presented in Section 5.2, the threat model presented in Section 3.2, and Theorem 2, one can see that the encryption mechanism leaks no information about the underlying health data to any PPT adversary modeling a group of unauthorized data users that colludes with the CSP. Therefore, our encryption mechanism is lightweight and secure. □

## 8. Performance Analysis

In this section, we analyze the performance of our LW-FGAC scheme by comparing its execution time, storage cost, and communication overhead with some existing ABE schemes in terms of both actual execution time and asymptotic complexity. The employed notations in the asymptotic analysis are given in Table 3.

In the asymptotic analysis, we considered three computational operations: exponential operation in $G_{1}$, exponential operation in $G_{2}$, and paring operation. As the other computational operations are significantly more efficient than the mentioned three operations, we ignore them in our analysis. Also, in measuring storage cost and communication complexity, we consider the size of elements in the groups $G_{1}$, $G_{2}$, and $Z_{q}$.

We implement our scheme by using an Ubuntu 18.04 laptop with an Intel Core i5-2410M Processor 2.3 GHz, 6 GB RAM using python Pairing-Based Cryptography (pyPBC) and hashlib libraries [45,46]. Also, we use the Type A pairings and SHA-1 algorithm. Moreover, in this section, we use And-gates access structure ($a_{1}\wedge \ldots \wedge a_{n}$) as the access control policy.

In the following, we describe our asymptotic and actual execution results. In our implementation, we assume that the number of leaf nodes in the access tree and the number of data users’ attributes are ranged between 10 to 100.

**Figure 7:**
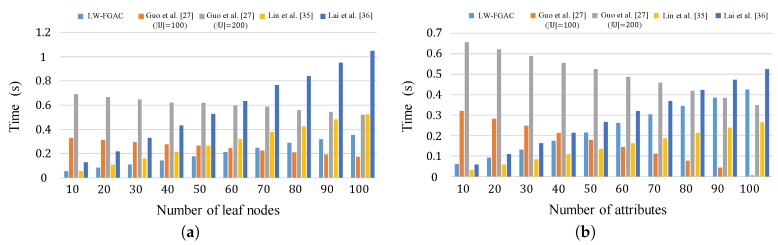
(**a**) Execution time of the encryption phase; (**b**) Execution time of the decryption phase.

**Figure 8:**
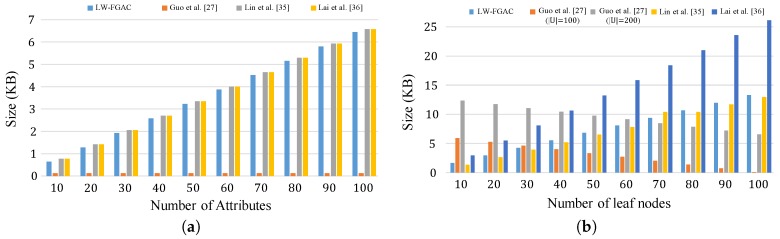
(**a**) Size of data users’ attribute secret-key; (**b**) Length of a ciphertext.

The actual execution times incurred by data owners and data users in the encryption and decryption phases are shown in Figure 7. As we see in part (a) of the figure, our encryption algorithm is significantly more efficient than the schemes presented in [27,35,36]. The mentioned fact is confirmed by the results given in Table 4. According to the figure, our scheme is more than 100 times faster than the schemes [27,35,36]. Also, as shown in Table 4, in [27], execution time is a function of the universal attribute set’s carnality, |U|. We measure its execution time when U∈{100,200}. One can see that this scheme is inefficient for large universal attribute sets, and data owners and data users have to perform a considerable amount of heavy computational operations. Also, Figure 8 and Table 5 compare the execution time of the encryption and decryption phases in LW-FGAC with the schemes presented in [27,35,36]. We see that the performance of our proposed scheme is acceptable in comparison with the other schemes.

**Figure 9:**
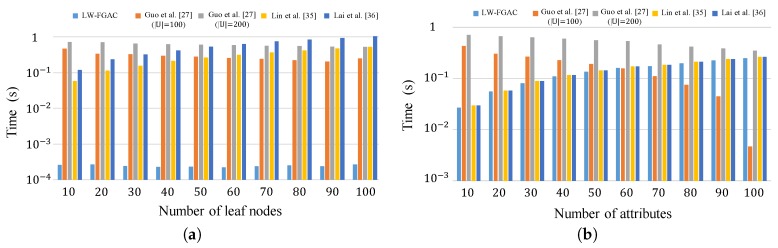
(**a**) Execution-time overhead on data owners in the encryption phase; (**b**) Execution-time overhead on data users in the decryption phase.

The storage overhead in our scheme and the schemes presented in [27,35,36] are given in Table 6 and Figure 9. Comparing the storage overhead in LW-FGAC with the others, one can see that the performance of LW-FGAC is acceptable. Also, we see that the data users’ secret-key size in [27] is significantly shorter than the others. However, the length of a ciphertext in [27] grows linearly with $\left|U|-\right|L_{T}|$, where |U| is the number of attributes in the system, and $|L_{T}|$ is the number of leaf nodes in the access tree associated with the ciphertext.

**Figure 10:**
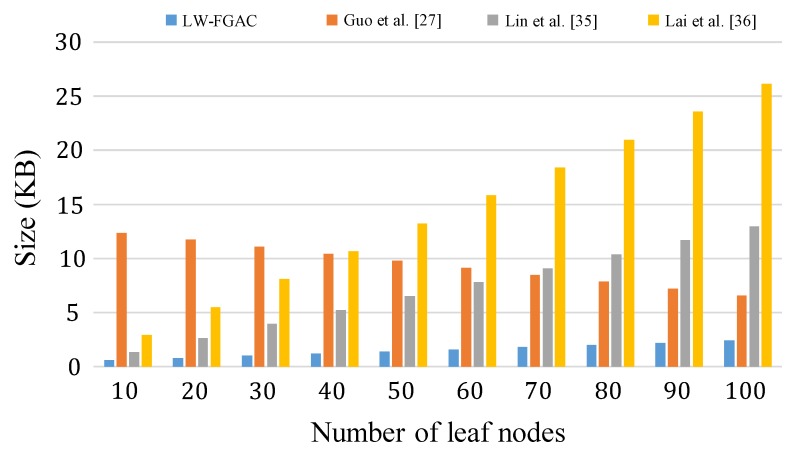
Communication overhead from data owners to the cloud.

Also, Figure 10 and Table 7 present the communication overhead from data owners to the cloud server. We see that our proposed scheme significantly reduces the overhead as in our scheme data owners just transmit lightweight partially encrypted data to the cloud server. However, in the other scheme, a complete ciphertext should be given to the cloud, which consumes more communication resources.

## 9. Conclusions

We designed a novel attribute-based cryptographic scheme called lightweight fine-grained access control (LW-FGAC) for cloud-based wireless body area networks (WBANs). In our proposed scheme, by performing very lightweight computational operations, a data owner can encrypt its data under an access tree defined by itself. Any data user that its attributes satisfy the access policy can decrypt the ciphertext. Also, in our designed system, the computational overhead on the data user side is very efficient, and most of the computations in the decryption phase are performed by the cloud service provider. We also provided the security definition for the new primitive, and we proved its security in the standard model under the hardness assumption of decisional bilinear Diffie-Hellman (DBDH) problem.

## Figures and Tables

**Table 1:** Comparison of Properties in Different ABE Schemes.

Schemes	KP/CP/DP-ABE	Lightweight	Flexible	Lightweight	Security Model
		Encryption Mechanism	Access Control	Decryption Mechanism	
[8]	ABE	No	No	No	Selective
[12]	DP-ABE	No	Yes	No	Selective
[24]	KP-ABE	No	Yes	No	Selective
[25]	CP-ABE	No	Yes	No	Selective
[26]	CP-ABE	No	No	No	Selective
[27]	CP-ABE	No	No	No	Selective
[28]	KP/CP-ABE	No	No	No	Adaptive
[29]	CP-ABE	No	Yes	No	Adaptive
[30]	KP-ABE	No	Yes	No	Selective
[31]	CP-ABE	No	Yes	No	Selective
[32]	CP-ABE	No	Yes	Yes	Adaptive
[33]	CP-ABE	No	Yes	Yes	Adaptive
[34]	DP-ABE	No	Yes	Yes	Selective
[35]	CP-ABE	No	Yes	Yes	Selective
[36]	CP-ABE	No	Yes	Yes	Adaptive
LW-FGAC	CP-ABE	Yes	Yes	Yes	Adaptive

**Table 2:** Notations Employed in The System Definition And Our Proposed Construction.

Notation	Description
λ	Security parameter of the system
U	Universal attribute set of the system
params	Public parameters of the system
MSK	Master secret-key of the HA
$Att_{u}$	Attribute set of a data user
$id_{u}$	Identifier of a data user
$SK_{O}$	Secret-key of a data owner
$PK_{O}$	Public-key of a data owner
*M*	A data file
T	An access tree
$PCT_{T}$	Partial ciphertext associated with an access tree T
$CT_{T}$	Ciphertext associated with an access tree T
$SK_{u}$	Attribute secret-key of a data user
$TK_{u}$	Decryption token generated by a data user in the decryption phase
*k*	Private-key generated by a data user in the decryption phase
$M^{\prime }$	Partial decrypted ciphertext

**Table 3:** Notations Employed in Our Asymptotic Analysis.

Notation	Description
$|Att_{u}|$	Carnality of a data user’s attribute set
|U|	Carnality of the universal attribute set
$|L_{T}|$	Number of leaf nodes in an access tree T
*S*	Carnality of a data user’s attribute set satisfying a given access tree
$T_{e_{1}}$	Exponential operation time in $G_{1}$
$T_{e_{2}}$	Exponential operation time in $G_{2}$
$T_{p}$	Pairing operation time
$l_{G_{1}}$	Size of an element in $G_{1}$
$l_{G_{2}}$	Size of an element in $G_{2}$

**Table 4:** Comparison of Computational Overhead on Data Owners and Data Users.

Schemes	Encryption	Decryption
Guo et al. [27]	$\left(2|U|-\right|L_{T}\left|+3\right)T_{e_{1}}$	$\left(2|U|-2|S|+3\right)T_{e_{1}}+3T_{p}+T_{e_{2}}$
Lin et al. [35]	$\left(2\right|L_{T}\left|+1\right)T_{e_{1}}$	$\left(|S|+2\right)T_{e_{1}}+T_{e_{2}}$
Lai et al. [36]	$\left(6\right|L_{T}\left|+4\right)T_{e_{1}}+2T_{e_{2}}$	$\left(|S|+4\right)T_{e_{1}}+T_{e_{2}}$
LW-ABKS	$T_{e_{2}}$	$\left(|S|+1\right)T_{e_{1}}+T_{e_{2}}$

**Table 5:** Computational Complexity in The Encryption And Decryption Phases.

Schemes	Encryption	Decryption
Guo et al. [27]	$\left(2|U|-\right|L_{T}\left|+3\right)T_{e_{1}}$	$\left(2|U|-2|S|+3\right)T_{e_{1}}+3T_{p}+T_{e_{2}}$
Lin et al. [35]	$\left(2\right|L_{T}\left|+1\right)T_{e_{1}}$	$\left(2|S|+1\right)T_{p}+\left|S\right|T_{e_{2}}+T_{e_{1}}$
Lai et al. [36]	$\left(6\right|L_{T}\left|+4\right)T_{e_{1}}+2T_{e_{2}}$	$\left(4|S|+2\right)T_{p}+2\left|S\right|T_{e_{2}}+2T_{e_{1}}$
LW-ABKS	$\left(2L_{T}+1\right)T_{e_{1}}+2T_{e_{2}}$	$\left(|S|+1\right)T_{e_{1}}+T_{p}\left(2|S|+1\right)+T_{e_{2}}$

**Table 6:** Storage Overhead.

Schemes	Key Size	Ciphertext Size
Guo et al. [27]	$2l_{G1}$	$\left(|U|-\right|L_{T}\left|+2\right)l_{G_{1}}$
Lin et al. [35]	$(|Att_{u}\left|+2\right)l_{G_{1}}$	$\left(\right|L_{T}\left|+1\right)l_{G_{1}}$
Lia et al. [36]	$(|Att_{u}\left|+2\right)l_{G_{1}}$	$\left(4\right|L_{T}\left|+3\right)l_{G_{1}}+2l_{G_{2}}$
LW-ABKS	$|Att_{u}|l_{G_{1}}$	$\left(2|L_{T}+1|l_{G_{1}}+2l_{G_{2}}\right)$

**Table 7:** Communication Overhead from Data Owners to the Cloud.

Schemes	Size of The Transmitted Data
Guo et al. [27]	$\left(|U|-\right|L_{T}\left|+2\right)l_{G_{1}}$
Lin et al. [35]	$\left(\right|L_{T}\left|+1\right)l_{G_{1}}$
Lia et al. [36]	$\left(4\right|L_{T}\left|+3\right)l_{G_{1}}+2l_{G_{2}}$
LW-ABKS	$\left(|L_{T}+1|l_{Z_{q}}+l_{G_{2}}\right)$
